# Enhanced Analgesic Properties and Reduced Ulcerogenic Effect of a Mononuclear Copper(II) Complex with Fenoprofen in Comparison to the Parent Drug: Promising Insights in the Treatment of Chronic Inflammatory Diseases

**DOI:** 10.1155/2014/505987

**Published:** 2014-06-19

**Authors:** Mariela Agotegaray, Fernanda Gumilar, Mónica Boeris, Ricardo Toso, Alejandra Minetti

**Affiliations:** ^1^INQUISUR, Departamento de Química, Universidad Nacional del Sur, Avenida Alem 1253, B8000CPB Bahía Blanca, Argentina; ^2^Laboratorio de Toxicología, Departamento de Biología, Bioquímica y Farmacia, Universidad Nacional del Sur, San Juan 670, B8000CPB Bahía Blanca, Argentina; ^3^Centro de Investigación y Desarrollo de Fármacos (CIDEF), Facultad de Ciencias Veterinarias, Universidad Nacional de La Pampa, Calle 5 y 116, 6360 General Pico, Argentina

## Abstract

Analgesic and ulcerogenic properties have been studied for the copper(II) coordination complex of the nonsteroidal anti-inflammatory drug Fenoprofen and imidazole [Cu(fen)_2_(im)_2_] (Cu: copper(II) ion; fen: fenoprofenate anion from Fenoprofen, im: imidazole). A therapeutic dose of 28 mg/kg was tested for [Cu(fen)_2_(im)_2_] and 21 mg/kg was employed for Fenoprofen calcium, administered by oral gavage in female mice to compare the therapeutic properties of the new entity. The acetic acid induced writhing test was employed to study visceral pain. The percentage of inhibition in writhing and stretching was 78.9% and 46.2% for the [Cu(fen)_2_(im)_2_] and Fenoprofen calcium, respectively. This result indicates that the complex could be more effective in diminishing visceral pain. The formalin test was evaluated to study the impact of the drugs over nociceptive and inflammatory pain. The complex is a more potent analgesic on inflammatory pain than the parent drug. Ulcerogenic effects were evaluated using a model of gastric lesions induced by hypothermic-restraint stress. Fenoprofen calcium salt caused an ulcer index of about 79 mm^2^ while the one caused by [Cu(fen)_2_(im)_2_] was 22 mm^2^. The complex diminished the development of gastric mucosal ulcers in comparison to the uncomplexed drug. Possible mechanisms of action related to both therapeutic properties have been discussed.

## 1. Introduction

Inflammation is a physiological and biological response of vascular tissues against harmful stimuli such as pathogens, damaged cells, or irritants. It is a process which can be classified as acute or chronic. In acute inflammation, an initial response of the body against the harmful stimuli is initiated by movement of plasma and leukocytes from the blood to the injured site, where a local inflammatory process is developed. On the other hand, chronic inflammation is characterized by the increment in the type of inflammatory cells present at the site of inflammation. This sustained physiological response in time causes simultaneous destruction and healing of the injured tissue [1]. Some examples of chronic inflammatory diseases are osteoarthritis, rheumatoid arthritis, degenerative joint disease, and ankylosing spondylitis. These disorders are associated with constant pain episodes and require the intake of nonsteroidal anti-inflammatory drugs (NSAIDs) as therapeutic agents in long-term treatment. These drugs exert the inhibition of inflammation by blocking the action of both isoforms of the cyclooxygenase enzyme (COX), known as COX-1 and COX-2. COXs convert arachidonic acid into prostaglandin H2 which is then metabolized into various prostaglandins, prostacyclins, and thromboxanes, which are the molecular mediators that start the inflammatory process [2]. The main problem related to these therapies is that they cause systemic side effects associated with gastrointestinal (GI), renal, and cardiovascular toxicity [3]. In particular, long-term NSAIDs therapies employed in the treatment of chronic inflammatory diseases produce GI toxicity due to the generation of gastric ulcers [4].

Transition metal coordination complexes represent a potential group of therapeutic agents with applications in medicinal biochemistry. Several researches have been developed in the employment of metal complexes for the treatment of many human diseases such as cancer, inflammatory disorders, and infectious diseases, among others [5].

Copper represents a transition metal with proven anti-inflammatory and analgesic properties [6], being a very important oligoelement implicated in inflammatory processes. Copper(II) complexes of NSAIDs as ligands generally consist of a group of molecules with enhanced therapeutic properties in comparison to the parent drug.

As previously mentioned, taking into account the GI toxicity associated with the intake of NSAIDs, the study of the gastric ulcerogenic effect of some copper(II) complexes with different ligands such as amino acids and NSAIDs has been developed using different methods. As a result, it has been found that the complexes tend to diminish the development of gastric lesions associated with the therapy [7].

Although there exists a lot of research in the synthesis and characterization of several copper(II)-complexes with NSAIDs [3], in the last years, the research dealing with this kind of compounds in terms of their therapeutic properties [8–12] has been limited. Preexisting therapies would greatly benefit from further in-depth study of these compounds, taking into account the low cost and simplicity of their synthesis and their improved therapeutic properties in comparison to the parent drug.

**Scheme 1:**
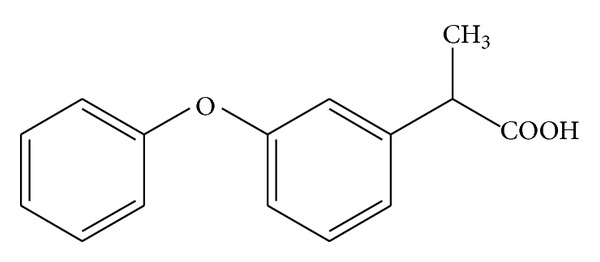
Molecular structure of Fenoprofen.

Fenoprofen [2-(3-phenoxyphenyl) propanoic acid] (Scheme 1) is a NSAID with analgesic and antipyretic properties, being useful in the treatment of chronic inflammatory diseases [13]. Because of its crystalline liquid properties, it is difficult to obtain new and derivative formulations from Fenoprofen. Our research group has carried out a thorough study of the complexation of Fenoprofen to copper(II) metal ion, being the first researchers to report the synthesis and characterization of copper(II)-fenoprofenate complexes [14, 15].

Ternary copper(II) complexes of Fenoprofen are promising therapeutic agents against inflammatory diseases because of their enhanced anti-inflammatory and analgesic effects compared to the parent Fenoprofen calcium salt [16, 17]. It was also demonstrated that two copper(II) complexes of Fenoprofen do not produce toxicological effects in mice after a subacute exposure, making this finding interesting for the potential employment of the formulations in possible clinical treatment.

**Figure 1:**
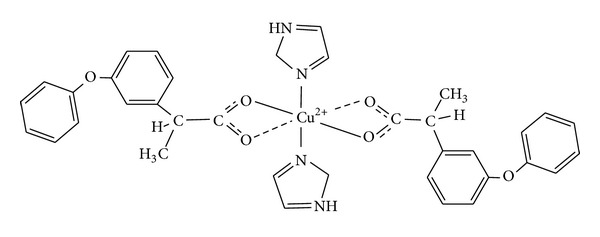
Molecular structure of the complex [Cu(fen)_2_(im)_2_].

In order to further contribute to the knowledge of the enhanced therapeutic properties of Fenoprofen copper(II) complexes, this study presents the antinociceptive and ulcerogenic activities of the first mononuclear copper(II)-fenoprofenate complex containing the bioactive molecule imidazole (im) (Figure 1). This coordination compound has been proved to present improved anti-inflammatory activity when compared to the uncomplexed Fenoprofen drug [13].

Therapeutic properties studied here were evaluated employing the acetic acid-induced writhing test and the formalin test in terms of the analgesic properties. Meanwhile, the ulcerogenic activity has been studied with the hypothermic-restraint stress inducing ulcers method, to compare with the uncomplexed commercial parent drug, Fenoprofen calcium salt. Possible mechanisms of action by which the complex could exert its enhanced therapeutic properties have been postulated.

## 2. Materials and Methods

The methodology followed to synthesize the complex [Cu(fen)_2_(im)_2_] has been performed as described in [17]. Briefly, a solution of 11.0 mg (0.160 mmol) of imidazole in 1 mL of acetone was added to another solution containing 50.0 mg (0.0400 mmol) of [Cu_2_(fen)_4_(dmf)_2_]^14^ (dmf: N,N′-dimethylformamide) in acetone. The resulting blue solution was kept under stirring for 30 minutes and then diffused with acetonitrile. After a few days at 4°C, the obtained blue crystals were washed with acetonitrile and air dried. The crystalline sample achieved by this synthesis procedure ensures the obtaining of a reproducible and pure compound suitable for biomedical applications* in vivo*. Full physicochemical characterization has been described in [17].

For the development of the study of the analgesic properties, healthy CF1 female mice, 8 weeks old, were employed. They were obtained from the colony of the animal facility from the Biology, Biochemistry, and Pharmacy Department and maintained under constant conditions of temperature (22 ± 1°C) and humidity (70%), in a 12 h light: 12 hours dark cycle (light on at 6:00) during all the experiment. According to the body weight (approximately 30 g), they were randomly divided into different groups of 5-6 animals which were acclimatized for a week before starting the experiment. All animals had free access to tap water and standard diet (Ganave, Ratas y Ratones, Alimentos Pilar S.A., Argentina) throughout the experiment.

The study of the gastric ulcerogenic properties has been carried out in healthy CF1 female mice of about 30 g provided by Facultad de Ciencias Veterinarias, Universidad Nacional de La Pampa. They were randomly divided into different groups of 6 animals; they had free access to tap water and were fed ad libitum with a standard diet (Cooperación, Asociación Cooperativas Argentinas, San vicente, Buenos Aires, Argentina). Four hours before the beginning of the bioassay, they were private of food.

The care and the handling of the animals were in accordance with the internationally accepted standard Guide for the Care and Use of Laboratory Animals [18] as adopted and promulgated by the National Institute of Health. All protocols were revised and approved by the affiliations where the studies have been carried out.

### 2.1. *In Vivo* Antinociceptive Activity

#### 2.1.1. Acetic Acid-Induced Abdominal Writhing

The test was performed as described by Collier et al. [19] and Fontenele et al. [20]. Nociception was induced by an intraperitoneal (i.p.) injection of 0.6% acetic acid solution (10 mL kg^−1^). Mice were orally treated by gavage with 28 mg kg^−1^ [Cu(fen)_2_(im)_2_], and 1 hour later the acetic acid was injected. The vehicle used for dissolution of complexes was 0.05% CMC-Na and 0.1% Tween 80. The control group received the vehicle and the positive group received 21 mg kg^−1^ Fenoprofen calcium salt. Each quantity of drug administered was equivalent to the therapeutic dose of 20 mg kg^−1^ of Fenoprofen, taking into account the good performance of this dose evaluated in previous work developed by us related to the anti-inflammatory properties of the drug and other derivative copper(II)-Fenoprofen complexes [15–17]. Immediately after the injection of acetic acid, each animal was isolated to an individual box to be observed for 20 minutes. The number of writhing and stretching was recorded. A writhe is indicated by abdominal constriction and stretching by full extension of hind limb. The results were expressed as inhibition percent in the number of writhing and stretching, calculated employing the formula [(1 − Tg)/Cg] × 100 with Tg being the number of writhing and stretching recorded in the treated group and Cg is the number of writhing and stretching recorded in the control group.

#### 2.1.2. Formalin Test

This test was carried out as described by Hunskaar and Hole [21]. Twenty microliters of 2.5% formalin were injected into the dorsal surface of the left paw of mice 1 hour after oral administration by gavage of 28 mg kg^−1^ [Cu(fen)_2_(im)_2_], 21 mg kg^−1^ Fenoprofen salt, or vehicle [0.05% CMC (carboxymethyl cellulose) and 0.1% Tween 80]. The time that animals spent on licking and biting the injected paw was recorded. On the basis of the response pattern described by Tjolsen et al. [22], two distinct periods of intensive licking activity were identified and scored separately. The nociceptive scores normally peaked 5 minutes after formalin injection (early phase) and 15–30 minutes after the injection (late phase).

### 2.2. *In Vivo* Ulcerogenic Activity

The bioassay was carried out in female mice employing the model of gastric lesions induced by hypothermic-restraint stress described by Levine [23] with some modifications. Animals were fasted for 24 hours before the experiment and received water* ad libitum*. They were orally* per os* administrated with each compound: [Cu(fen)_2_(im)_2_], 28 mg kg^−1^; Fenoprofen calcium salt, 21 mg kg^−1^; CuSO_4_, 12 mg kg^−1^. The control group received only the vehicle (0.05% CMC and 0.1% Tween 80). Each dose was calculated in order to obtain a final therapeutic dose of Fenoprofen estimated in 20 mg kg^−1^; for CuSO_4_, the dose administered contained the same copper(II) quantity of the corresponding coordination complex studied. The purpose of including CuSO_4_ in the study is to evaluate the role of free copper(II) ions.

After one hour, mice were immobilized in cages with individual cylindrical cells of 2.2 cm diameter and 10 cm in length and immersed in water at 22°C to the xiphoid cartilage for 4.5 hours.

The stomachs of euthanized animals by cervical dislocation were insufflated, removed, and fixed in formol 10% solution. After 6 hours they were opened by the mayor curvature and photographed. Digitalized photographs were analyzed by an image analyzer in order to determine the ulcerated area of each stomach. The total damaged stomach surface (mm^2^) was expressed as the ulcer index (UI).

### 2.3. Statistical Analysis

Data obtained from each study were tested using one-way ANOVA followed by Student's *t*-test when differences between groups were detected. Probability values less than 0.05 were considered to be significant.

## 3. Results

**Figure 2:**
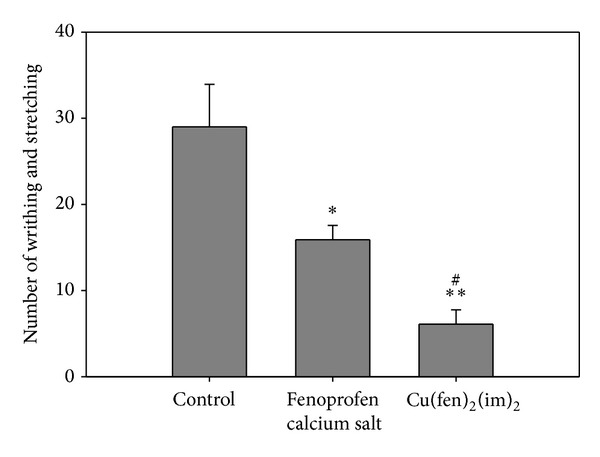
Effects of Fenoprofen calcium salt (21 mg kg^−1^) and [Cu(fen)_2_(im)_2_] (28 mg kg^−1^) on the acetic acid test. Both compounds were administered by oral gavage 1 hour before the acetic acid injection (i.p.). The number of writhing and stretching per mouse was counted over a 20-minute period. Values are expressed as means ± SEM; *n* = 9 − 10. *P < 0.05 and **P < 0.01 with respect to the control group administered with the vehicle. ^#^ *P* < 0.01 with respect to the Fenoprofen group.

Results obtained from acetic acid-induced writhing are shown in Figure 2.

When acetic acid test was analyzed, the one-way ANOVA reveled significant differences among the groups, *F*(18.27) = 24.5, *P* < 0.001. Fenoprofen calcium salt and the complex showed inhibitory effects on the writhing response induced by acetic acid. Treatment with [Cu(fen)_2_(im)_2_] and Fenoprofen salt significantly diminished the acetic acid-induced writhing response compared with the control group (*P* < 0.01 and *P* < 0.05, resp.). The percentage of inhibition was 78.9% and 46.2% for the complex and the uncomplexed parent drug, respectively. It is remarkable the diminishing in writhing and stretching caused by [Cu(fen)_2_(im)_2_] in comparison to Fenoprofen calcium salt (*P* < 0.01). This result indicates an excellent performance for the complexation of the drug to copper(II) ions related to the analgesic effect.

**Figure 3:**
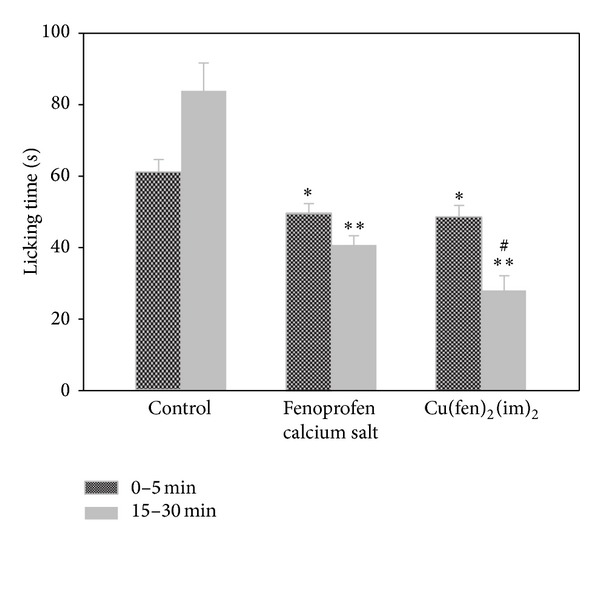
Effects of Fenoprofen calcium salt and [Cu(fen)_2_(im)_2_] on the formalin test. Values are expressed as the mean ± S.E.M. of time that mice spent on licking and biting the injected paw in the early phase (dark grey bar) and the late phase (gray bar). *n* = 9-10. *P < 0.05 and **P < 0.01, respectively, to the control group administered with the vehicle. ^#^ *P* < 0.01, respectively, to the group administered with the Fenoprofen calcium salt.

Figure 3 shows results obtained for the study of the analgesic properties evaluated by the formalin test in mice. In this test, the one-way ANOVA revealed significant differences among the groups, *F*(2.33) = 6.4, *P* < 0.01 for the early phase, and *F*(2.33) = 32.9, *P* < 0.001 for the late phase. The results obtained (Figure 3) showed that the time spent on licking the injured paw was significantly attenuated in the early phase by both Fenoprofen calcium salt and the complex in comparison to the control group (*P* < 0.05). In the second phase, the inhibition was more significant for both compounds (*P* < 0.01). Anyway, [Cu(fen)_2_(im)_2_] showed a marked inhibition of licking responses in the late phase with respect to the group administered with the Fenoprofen calcium salt (*P* < 0.01). Therefore, the complex presented a stronger effect than the corresponding calcium salt in the inhibition of pain during the second phase of the assay associated with the inflammatory pain.

Table 1 shows the ulcerogenic effect expressed as ulcer index in mice induced by Fenoprofen calcium salt, free copper(II) ions (administered as CuSO_4_), and the complex [Cu(fen)_2_(im)_2_].

Regarding the study of the ulcerogenic effect of the compounds, the employment of hypothermic restraint model was introduced as an external stimulus for ulcer generation. All the compounds studied incremented ulceration when administered in comparison to the control group. Fenoprofen calcium salt resulted in the most ulcerogenic compound with respect to the control (*P* < 0.001). This fact is expectable taking into account the gastrointestinal toxicity associated with the intake of NSAIDs.

Administration of free copper(II) ions was implemented to evaluate the role of copper(II) in ulcerogenesis, finding that it produced the lowest ulceration index, indicating that it would not cause severe ulcerogenic damage. On the other hand, the copper(II)-fenoprofenate-imidazole complex significantly diminished the damage to gastric mucosal in comparison to the uncomplexed parent drug (*P* < 0.01), as can be observed in the ulcer indexes reported in Table 1 obtained for the complex in comparison to Fenoprofen calcium salt. Ulceration caused by [Cu(fen)_2_(im)_2_] resulted in 3.6 times lower than the one caused by the uncomplexed commercial drug. By this way, it is clearly demonstrated that complexation of Fenoprofen to copper(II) ion reduced the ulcerogenic effect of the free NSAID molecule.

**Figure 4:**
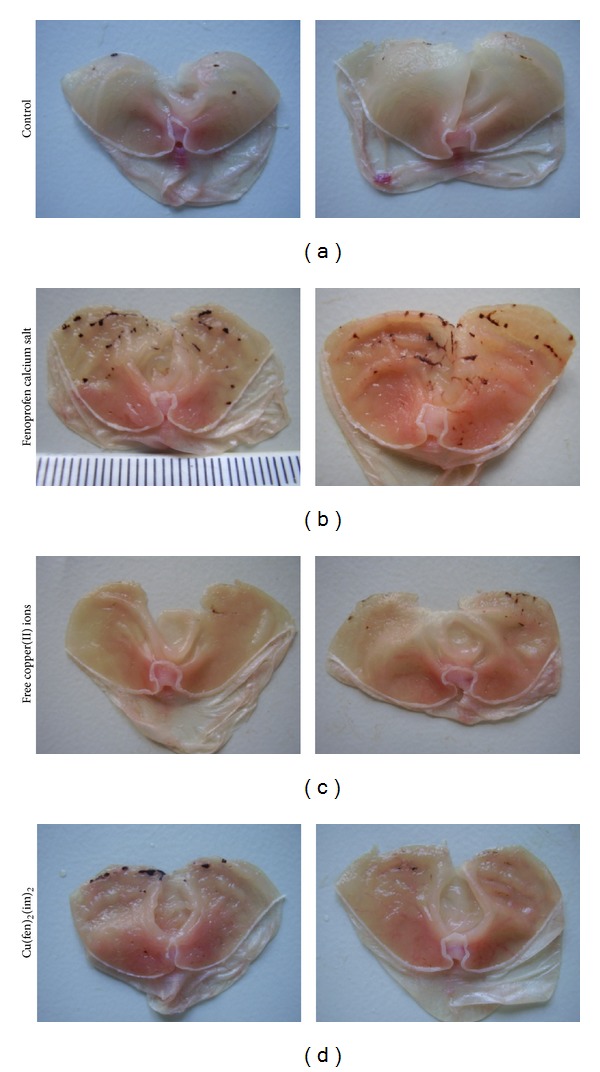
Mice stomachs with gastric lesions induced by Fenoprofen calcium salt, free copper(II) ions, and the coordination copper(II) complex (in comparison to the control group). The presence of milder injuries in the stomachs belonging to the group treated with the complex rather than those administered with calcium Fenoprofen is evident.

Figure 4 shows representative photographs of mice's stomachs of each group administered with Fenoprofen calcium salt, free copper(II) ions, and [Cu(fen)_2_(im)_2_]. The decrease of ulcerated lesions in stomachs of mice treated with the complex is observable.

## 4. Discussion 

### 4.1. Analgesic Activity

Acetic acid-induced writhing is a visceral pain model employed to evaluate antinociceptive activity [24]. Intraperitoneal administration of acetic acid causes the release of prostaglandins and sympathomimetic mediators [25] which are activators of the nociceptive mechanism. The complex studied significantly reduced the number of abdominal constrictions and stretching of hind limbs in mice. The percentage of inhibition was 78.9% for [Cu(fen)_2_(im)_2_] and 46.2% for Fenoprofen salt. The visceral analgesic action of the complex was significantly more potent than Fenoprofen calcium salt at the same therapeutic Fenoprofen dose (20 mg kg^−1^). In this way, [Cu(fen)_2_(im)_2_] presented a stronger analgesic activity for visceral pain. Okuyama et al. have studied the effect of free copper(II) in analgesia [26]. Through the acetic acid-induced writhing test, they have found that copper salts [Cu(chloride)_2_ and Cu(acetate)_2_], even when presenting analgesic activity, are not as effective as the corresponding copper(II) complexes with NSAIDs such as indomethacin, aspirine, and niflumic acid.

The formalin test is employed as a model for nociceptive pain [27, 28] being useful for the evaluation of nociceptive and inflammatory pain. Animals present two distinct nociceptive behaviour phases: the early phase (phase 1) initiates immediately after formalin injection and lasts about 3–5 minutes while the late phase (phase 2) is initiated 15–20 minutes after injection and lasts about 20–40 minutes. The development of phase 1 results from the chemical stimulation of nociceptors and nerves by formalin. The nociception produced in phase 2 is a result of chemical insult resulting in tissue damage [24] with the concomitant generation of inflammation mediators [22]. NSAID agents have little effect on the early phase but they reduce the nociceptive behavior of licking and biting in the late phase [21].

As expected, Fenoprofen calcium salt and [Cu(fen)_2_(im)_2_] presented little effect on the early phase of the formalin test, taking into account the effect of NSAIDs, but it reduced the nociceptive behaviour in the late phase. This reduction is associated with the inhibition of COX-2 with concomitant blockage of prostaglandin secretion. The complex showed remarkable inhibition of licking responses in the late phase in comparison with the Fenoprofen salt.

### 4.2. Mechanisms Proposed for Enhanced Analgesic Properties of the Complex

**Scheme 2:**
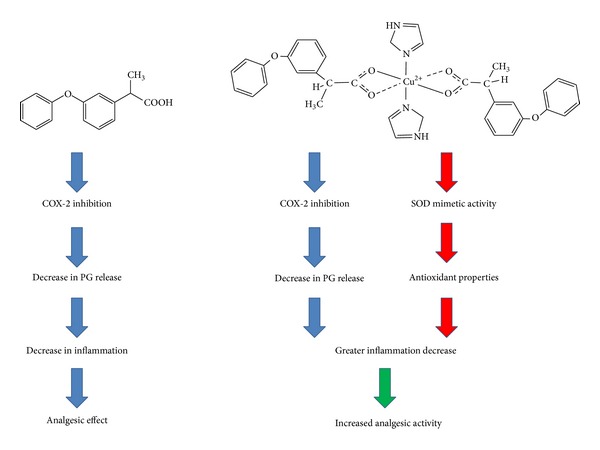
Proposed mechanism for the incremented analgesic activity of [Cu(fen)_2_(im)_2_] in comparison to the uncomplexed commercial parent drug (COX-2: cyclooxygenase-2; PG: prostaglandins; SOD: superoxide dismutase).

In previous work by this research team, it has been demonstrated that [Cu(fen)_2_(im)_2_] exerts enhanced anti-inflammatory properties in comparison with Fenoprofen as well as presenting superoxide dismutase (SOD) mimetic activity [17]. SOD enzyme is a copper-zinc metalloprotein encouraged to catalyze the conversion of the oxidative superoxide anion (O_2_^−·^), diminishing in this way the oxidative stress present in the pathophysiology of inflammatory processes [29]. The SOD mimetic activity imparts antioxidant properties to the complex, being this characteristic very important in the inflammatory process, as it contributes to diminish inflammation. This property, added to the known effect of NSAID in blocking inflammation by inhibiting COX-2, would increase the anti-inflammatory activity of the coordination complex versus the free NSAID. This activity would be responsible for the better performance regarding the decrease in nociceptive pain in the late phase of the formalin test compared to Fenoprofen calcium salt, making [Cu(fen)_2_(im)_2_] a better analgesic drug.  Scheme 2 illustrates the proposed mechanism by which the complex exerts increased analgesic properties in comparison to Fenoprofen.

The presence of copper in the complex may largely explain the enhanced therapeutic properties of [Cu(fen)_2_(im)_2_]. Nevertheless, other plausible mechanisms of action exist by which the copper complex could increment the antinociceptive effect of Fenoprofen: (I) copper complexes could act as transporters of copper(II) to the site of inflammation, where the metal ion is necessary as a cofactor of some enzymes such as superoxide dismutase [30]. The incremented bioavailability of copper(II) ion by the copper(II) coordination complex and its SOD mimetic activity would be two factors implicated in the reduction of inflammation which would concomitantly diminish the nociceptive effect. (II) Complexed forms of copper(II) would facilitate absorption and tissue distribution of copper as well as the associated ligands, thus incrementing their bioavailability [31]. Taking into account this possible mechanism, the copper(II)-Fenoprofenate-imidazole complex studied here could be acting as a moiety that is more absorbable than the uncomplexed parent drug, incrementing Fenoprofen bioavailability, thus potentiating its anti-inflammatory activity and, in this way, the antinociceptive effect.

It is important to mention that oral administration of the complex would not interfere with the analgesic activity, taking into account that the therapeutic effect of the complex is higher compared to that of the uncomplexed Fenoprofen. If stomach digestion promoted dissociation of the complex, the observation of enhanced analgesic activity would not be possible, considering that the Fenoprofenate anion concentration is equivalent to the amount in the Fenoprofen calcium salt and [Cu(fen)_2_(im)_2_] doses studied.

### 4.3. Ulcerogenic Effect

NSAIDs cause GI mucosa damage by several mechanisms: direct irritation to the epithelium, reducing mucosa blood flow, interfering with the normal repair of superficial injury, or inhibiting prostaglandins synthesis by blocking the activity of cyclooxygenase enzyme (COX) [32]. There exist two COX isoenzymes: COX-1, a house-keeping enzyme, and COX-2, primarily induced by inflammatory stimuli in various cells. NSAIDs exert their anti-inflammatory activity inhibiting COX-2 isoenzyme, while the downregulation of COX-1 is associated with gastric ulceration [33].

Hypothermic-restraint stress inducing ulcers method is commonly used for the evaluation of antiulcer activity in animals [34]. The possible mechanism involved in the induction of ulcers could be the increase of histamine release which enhances acid secretion [35] and reduces mucus production [36]. Alterations in gastric mucosal microcirculation and abnormal motility are also proposed as pathogenic mechanisms caused by stress-induced gastric mucosal lesions [37]. Nevertheless, the most important factor in the genesis of stress ulcer is the vagal over-activity that increases gastric acid secretion.

Taking into account the mechanism proposed for ulcerogenesis caused by the NSAIDs, it seems fitting to analyze the possible mechanism exerted by the copper(II)-fenoprofenate complex to protect the stomach against mucosal damage. Fenoprofen produces a great area of mucosal damage, probably by the mechanisms previously described for all NSAIDs in general. Even when [Cu(fen)_2_(im)_2_] increases ulceration in comparison to the control group, it exhibits a lower ulcerogenic effect in comparison to Fenoprofen calcium salt. Thus, the lower ulcerogenic action of [Cu(fen)_2_(im)_2_] in comparison to uncomplexed parent drug could be due to various reasons: (I) the association between copper(II) and Fenoprofen would protect the gastric epithelium against the direct irritation caused by free Fenoprofen; (II) Its antioxidant properties, due to the superoxide dismutase enzyme (Cu-SOD) mimetic activity, could be favourable to diminish lesions associated with tissue injury. As mentioned above, [Cu(fen)_2_(im)_2_] has the property of being a mimetic of the (Cu-SOD). This enzyme takes part in many oxidative stress processes because it catalyzes the scavenging of superoxide anion O_2_^−·^ acting as a relevant physiological antioxidant; (III) copper(II) complexes present more lipophilic character than the corresponding uncomplexed ligands and have proven gastric antisecretory activity [26]. The higher lipophilicity of [Cu(fen)_2_(im)_2_] would increase the absorption of this complex by the stomach cells, thus facilitating its antisecretory activity in situ, and this would result in decreased gastric acidity.

## 5. Concluding Remarks

In this work, the antinociceptive and the ulcerogenic properties of [Cu(fen)_2_(im)_2_] have been studied. The copper(II) complex presented enhanced analgesic activity when compared to Fenoprofen due to its potent anti-inflammatory activity. It also exerted a lower ulcerogenic action than the parent drug, diminishing the development of gastric mucosal ulceration. With these results and based on our previous experience, it is possible to postulate that both the enhanced analgesic activity compared to Fenoprofen calcium salt and the reduced ulcerogenic effect of the complex studied here could be attributed to the antioxidant activity imparted by the presence of copper(II) ion in its structure. The present study permits to establish that this complex is a promising alternative therapeutic agent against conventional therapy with NSAIDs, not only because of its biomedical properties but also because of its easy and cheap obtention. The presence of copper(II) in the molecular structure of the complex is responsible for enhancing the therapeutic properties of uncomplexed Fenoprofen. This metal ion plays a crucial role in inflammation and pain associated processes. [Cu(fen)_2_(im)_2_] could be applied as a therapeutic agent in the medical treatment of chronic inflammatory diseases because it prevents side effects associated with the therapy with NSAIDs such as ulcerogenesis, and because it is a more potent analgesic agent than Fenoprofen.

## Figures and Tables

**Table 1:** Ulcerogenic effect caused by the tested compounds in mice.

Compound	Ulcer index (UI)/mm^2^
Control	3.00
Fenoprofen calcium salt	79.0
Free copper(II) ions	11.5
[Cu(fen)_2_(im)_2_]	22.2
